# Advanced SBAS-DInSAR Technique for Controlling Large Civil Infrastructures: An Application to the Genzano di Lucania Dam

**DOI:** 10.3390/s18072371

**Published:** 2018-07-21

**Authors:** Marco Corsetti, Fabrizio Fossati, Michele Manunta, Maria Marsella

**Affiliations:** 1 Department of Civil, Constructional and Enviromental Engineering (DICEA), Sapienza Università degli Studi di Roma, Via Eudossiana 18, 00184 Rome, Italy; faber.fossati@gmail.com (F.F.); maria.marsella@uniroma1.it (M.Mar.); 2 Institute for Electromatic Sensing of the Enviroment-National Research Council (IREA-CNR), Via Diocleziano 328, 80124 Naples, Italy; manunta.m@irea.cnr.it

**Keywords:** dam, monitoring, SBAS-DInSAR, FEM modelling, subsidence, consolidation, geotechnical measurements

## Abstract

Monitoring surface deformation on dams is commonly carried out by in situ geodetic surveying, which is time consuming and characterized by some limitations in space coverage and frequency. More recently microwave satellite-based technologies, such as advanced-DInSAR (Differential Synthetic Aperture Radar Interferometry), have allowed the integration and improvement of the observation capabilities of ground-based methods thanks to their effectiveness in collecting displacement measurements on many non-destructive control points, corresponding to radar reflecting targets. The availability of such a large number of points of measurement, which are distributed along the whole structure and are characterized by millimetric accuracy on displacement rates, can be profitably adopted for the calibration of numerical models. These models are implemented to simulate the structural behaviour of a dam under conditions of stress thus improving the ability to maintain safety standards. In this work, after having analysed how advanced DInSAR can effectively enhance the results from traditional monitoring systems that provide comparable accuracy measurements on a limited number of points, an FEM model of the Genzano di Lucania earth dam is developed and calibrated. This work is concentrated on the advanced DInSAR technique referred to as Small BAseline Subset (SBAS) approach, benefiting from its capability to generate deformation time series at full spatial resolution and from multi-sensor SAR data, to measure the vertical consolidation displacement of the Genzano di Lucania earth dam.

## 1. Introduction

The safety of large civil structures is assured not only by reliable design and construction approaches but also through the constant control of its behaviour at different operational stages. Specifically, it is important to measure the real values of the displacements to be compared with those expected and estimated during the design stage. Displacement can be compared with the results of numerical modelling, in order to identify the presence of anomalies and avoid reaching Limit States. Traditional topographical techniques provide measurements on a limited number of control points that are not usually enough to describe the overall deformation pattern and often are not even adequate to verify the effectiveness of the structural models. On the contrary, the use of automated total stations, terrestrial remote sensors (Terrestrial Laser Scanning) and satellite-based techniques allows a systematic and distributed control of any structural movements, without needing to access the structure [1,2,3,4].

Nowadays, many civil structures are monitored only superficially or even not monitored at all (i.e., in some cases only visual inspection is adopted to control structure behaviour). The lack of a proper monitoring system increases the risk connected to potential critical events and it does not permit an adequate level of knowledge of the structural behaviour over time. This absence of information often leads to operations to be carried out only in an emergency phase as well as the difficulty in detecting the origin of any failure.

**Figure 1:**
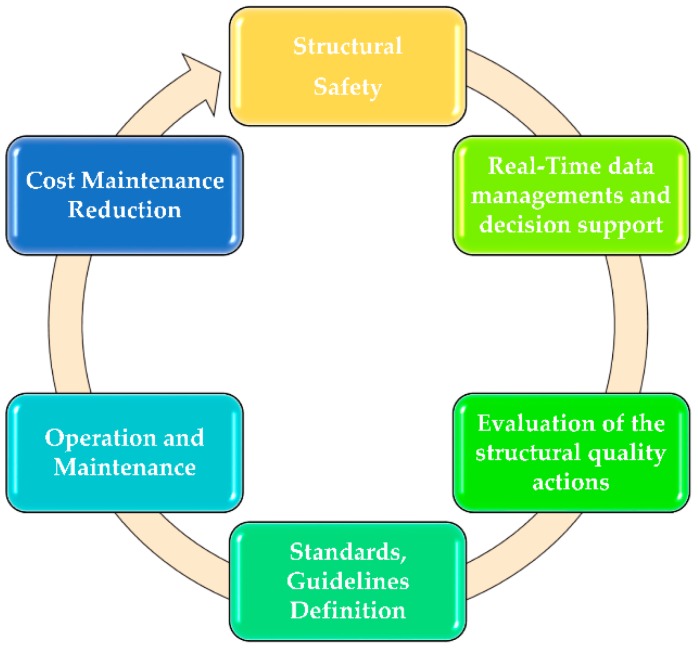
Diagram on the optional monitoring approach.

By adopting a systematic and integrated monitoring approach (Figure 1), based both on ground and satellite instruments, many advantages can be gained as regards safety, the optimization of maintenance and management costs.

Among the available satellite techniques, Differential Synthetic Aperture Radar (SAR) Interferometry (DInSAR) represents one of the most suitable technologies to monitor deformations affecting dams. Indeed, DInSAR technique allows the retrieval of sub-centimetric ground displacements exploiting the phase difference (interferogram) between to SAR images acquires in different times. Originally proposed to investigate surface displacements related to natural events such as earthquakes and volcanic activity, more recently several advanced DInSAR techniques have been developed to investigate the temporal behaviour of the detected displacements by benefitting of large SAR datasets composed of tens or hundreds of SAR acquisitions acquired over more 15 years. One of the advanced DInSAR techniques that has demonstrated its capability to retrieve surface displacements with centimetric to millimetric accuracy is the Small Baseline Subset (SBAS) approach [5,6]. The SBAS algorithm relies on a proper selection of a large number of SAR data pairs. These pairs are used to generate small baseline differential interferograms to mitigate the noise (i.e., the decorrelation phenomena) affecting the interferograms and to maximize the number of reliable points of measurement. For each coherent measure point, displacement time series and mean deformation velocity are retrieved with an accuracy of about 5–10 mm for the single deformation measurement and 1–2 mm/year for what concerns the mean deformation velocity information [7,8,9].

In this work, we focus on the exploitation and integration of deformation measurements retrieved through the SBAS algorithm with in situ information within a Finite Element Model analysis. The proposed methodology can be efficiently used for assessing the behaviour of a dam during life-cycle Moreover, the analysis of the displacement time series derived from the SBAS-DInSAR technique allows us to perform back analyses also in the periods in which other monitoring data were not available. Finally, benefitting from very long-time series covering the 1992–2007 time period, we can effectively set up a reliable numerical model that provides detailed information of the status of the infrastructure.

## 2. Large Dam Monitoring

**Figure 2:**
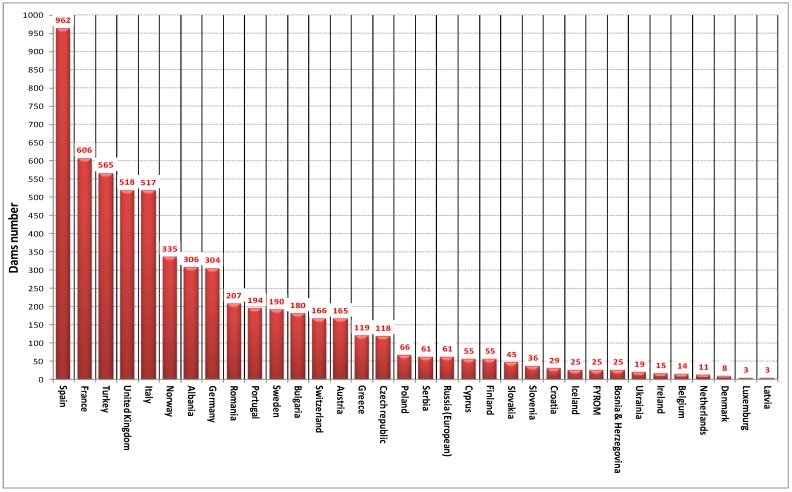
Dam distribution in the European countries.

The International Commission on Large Dams (ICOLD) maintains a Register of Dams in the World. For a dam to be considered large and be included in the register, it must have a height of 15 m or 10 to 15 m and store more than 3 million cubic meters of water in the reservoir. The world data as of 2000 indicates that there are about 50,000 large dams in operation and 6000 in Europe (Figure 2).

**Figure 3:**
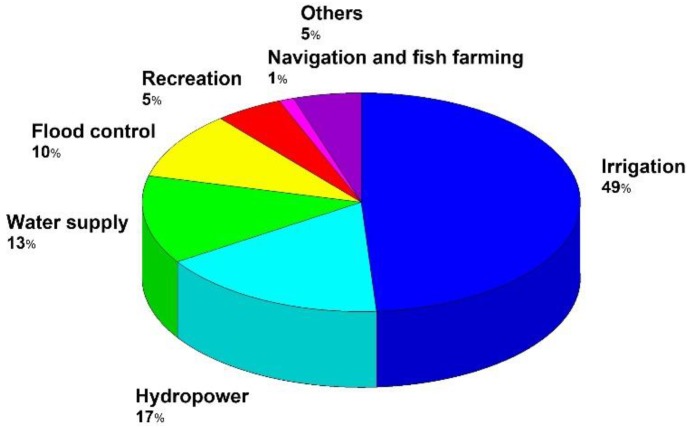
Distribution for dams associated to different usage.

Most of these are single-purpose dams (71.7%) but there is a growing number of multipurpose dams (28.3%). Today, irrigation is the most common purpose. Figure 3 shows the distribution for each purpose.

The average life expectancy of a non-strategic dam is about 50 years. Presently 25% of the operational dams are more than 50 years old (i.e., in Italy the average is over 60 years) and this will become 85% by the year 2020. Furthermore, a great number of dams are monitored using obsolete instruments that may be not reliable or accurate enough.

Dams are very old structures that should be carefully monitored by enforcing and enlarging the observation capability and the accuracy of the existing monitoring systems.

A relevant aspect of the dam monitoring is that, in most countries, dam owners and operators have to follow the guidelines developed by their own National Committees or those published by ICOLD (International Commission on Large Dams). In addition, dam owners have established their own standards/specifications based on their own experience and as a result there is no common standard between countries.

Generally, the accuracy of the measured displacement (at a 95% confidence level) should be at least 3 times smaller than the maximum expected displacements over the observation time interval. Each dam requires different monitoring accuracy depending on its dimensions, material type, interaction with foundation bedrock and so forth. Concrete dams and structures of hydro-electric power plants require monitoring measurements which are approximately 5 times more accurate than the ones collected on embankment dams and slopes surrounding reservoirs.

Table 1 lists the expected accuracies and the recommended types of monitoring technologies (geodetic and/or geotechnical) to be used during the normal behaviour of the structures. However, once irregularities in behaviour are observed, there is no limit to how much the accuracy should be increased, other than that of costs. High accuracy measurements can be used to analyse the causes and mechanism of unexpected deformations.

Table 2 illustrates some of the traditional types of instruments used to control embankment and concrete dams. Due to the differences between embankment and concrete dams (structural behaviour and displacements entities), a monitoring scheme cannot be designed in the same manner for both. With concrete dams, it is important to observe the behavioural trends in both the elastic and plastic (permanent) deformation. This analysis implies comparing the measured deformation with that predicted in normal behaviour. However, with embankment dams, permanent deformation trends should be intimately monitored for any sign of abnormality.

The minimal configuration of the monitoring system requires instruments be located where:

- the maximum deformations are expected;
- the deformation is small but is considered to be critical to the safety of the dam; and
- a deformation phenomenon is to be observed.

Nowadays, developments in technology allow us to utilize new advanced technologies, in particular geodetic/remote sensing sensors (ground-based and space) and geotechnical/structural instruments (e.g., tiltmeters, extensometers and strainmeters). Geodetic methods supply information on the absolute and relative displacements (changes in coordinates) from which displacement and strain fields for the monitored object may be derived. Thus, geodetic surveys supply global information on the behaviour of the object under investigation. Among the available geodetic and geotechnical/structural technologies, very few, if any, sensors can fully satisfy the monitoring criteria as a stand-alone system. Therefore, in most cases, various techniques must be combined into an integrated monitoring system.

Monitoring needs vary over the different phases occurring during the life of a dam. These phases typically include design, construction, first reservoir filling, long-term (normal operations) and dealing with unexpected performance (critical events). Instrumented monitoring can be a very effective tool for obtaining the information needed during these different phases. In addition to monitoring the performance of the dam, instrumentation data can also be valuable for legal or research considerations.

## 3. Case Study: Genzano di Lucania Dam

### 3.1. The Genzano di Lucania Dam

**Figure 4:**
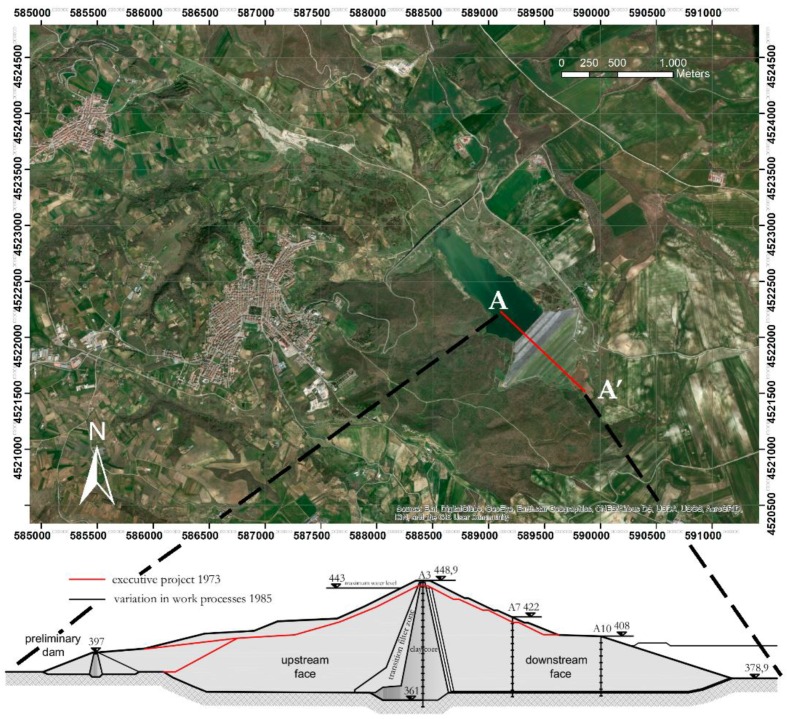
(**a**) Ortophoto of the Genzano di Lucania reservoir; (**b**) main cross section of the dam (modified in December 1985 from the initial configuration indicated by red lines.

The Genzano dam (Figure 4) is an 88 m-high zoned earthfill dam. Between the core and the embankments there is a transition upstream zone and double-layer filters on the downstream. The cofferdam is made of rockfill material with a low permeability core.

The dam is founded on the blue-grey clay formation which is typical of the Ofanto River valley. The construction project [10] is dated December 1973. The dam was built between July 1979 and January 1993.

During this period, due to the Irpinia earthquake (23 November 1980) and to the issuing of the new Italian building code (D.M.LL.PP. 24-03-1982—“Technical standards for the design and construction of dams”), a modification of the project was required (December 1985) to change the geometry of the cross section. The main changes were the elevation of the dam crest, from 445 to 448.9 m a.s.l. and the increase of the width of crest dam from 8 to 10 m [10] (Figure 4b).

The reservoir from the construction of the dam until today it has never been filled.

### 3.2. Geological Setting

The Genzano di Lucania Dam (Figure 4) is located in the Bradano River Valley (Potenza province, Basilicata region, Italy).

The geology of this territory is characterized by the presence of Subappennine Argilaceous layers datable to the Upper Pleiocene, the largest water courses are the Bradano, Basentello and Fiumarella rivers. A line of clayey hills gently falling towards the lake characterize the basin geomorphology. Immediately below the dam, the Bradano River flows over an ancient calcareous substrate dating from the Cretaceous and forms a characteristic “gravina” (ravine), a sort of depression dug out from the rock by the erosive action of the stream water.

The surface terrain in the catchment basin, is the product of the sedimentary cycle of the Bradanic trench and is formed, mainly, from the oldest to the most recent of:

- blue or yellowish altered clays;
- limestone quartz yellowish sands; and
- polygenic conglomerates with pebbles also made by crystalline rocks and interbedded with lenses of sandy and clay.

As envisaged in the preliminary plan, geotechnical investigations consisting of surveys, trenches and surface geological surveys evidenced, at the foundation section, the presence of blue clays at depths varying between 10 and 20 m in a layer of 300 m. The clays are covered by a thin layer of altered brunette clay.

### 3.3. Hydrological Setting of Genzano di Lucania Dam

The hydrographic network of the Basilicata, region Italy (is based on five main rivers (Bradano, Basento, Cavone, Agri and Sinni) flowing into the Ionian Sea, one (Ofanto) flowing into the Adriatic Sea and two (Sele and Noce) flowing into the Tyrrhenian Sea. Besides the rivers, there is an extensive network of small waterways and numerous springs. The hydrographic network of the Basilicata region is regulated by a complex hydraulic system composed of dams, crosses, spring and groundwater collectors, supply networks and distribution systems. This infrastructure system was developed between 1950 and 1960 and later integrated through the construction of new infrastructures, in order to increase water supply to industrial plants and hydroelectric power to the neighbouring regions, Puglia and Calabria.

### 3.4. Instrumental Monitoring Data

**Figure 5:**
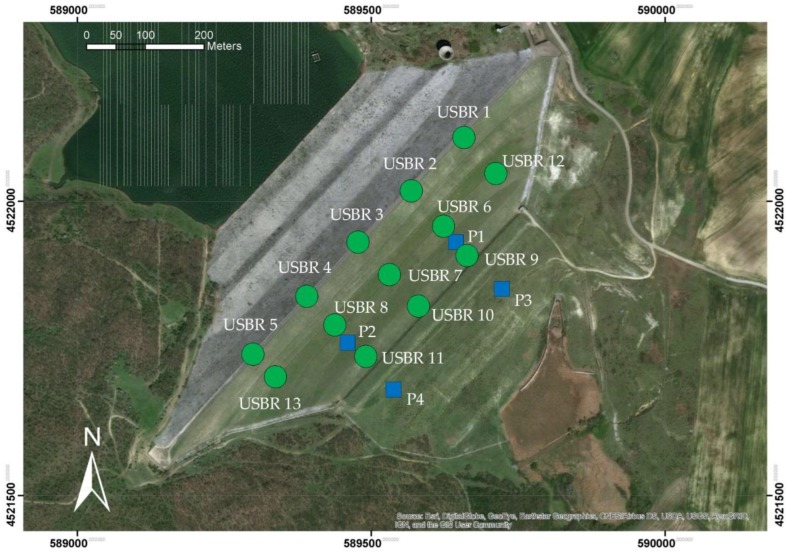
Position of the installed extensometers USBR model (green circles) and piezometers (blue squares) on the dam.

The deformation monitoring system of the dam includes different sensors (Figure 5):

- 51 vibrating wire piezometers;
- 18 pressure cells;
- 9 open standpipe piezometers of which 4 located on the downstream face (P1, P2, P3 and P4); and
- 13 extensometers USBR model (U.S. Bureau of Reclamation): 5 located inside the core (USBRs 1 and 5) and 8 inside the downstream embankment.

Reservoir level, precipitation and air and water temperature are also recorded.

**Figure 6:**
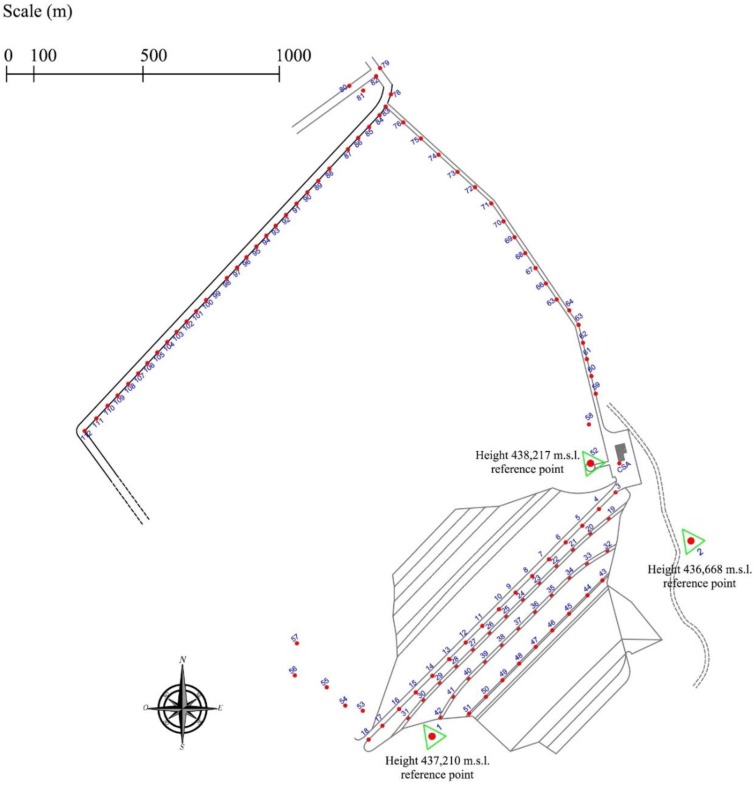
Levelling surveying: location of the benchmarks.

The measurements have been performed through high precision level Leica NA 3000 model from Leica geosystem, by automatic take-over on invar levelling staffs. The benchmarks number 1, 2 and 52 (on the control tower) represent the reference points for the altimetry measurements. The points 1 and 52 stability have been checked through the height differential with the point 2 as it is positioned on a geologically stable ground, as illustrated in Figure 6. 

The levelling measurements are repeated every three months. The control points are located on the dam as indicated in Figure 6:

- 16 at the crest of dam (from 3 to 18);
- 13 at the first downstream berm (from 19 to 31);
- 11 at the second downstream berm (from 32 to 42); and
- 9 at the third downstream berm (from 43 to 51);

Three points used as reference (point 1, point 2 and point 52) (Figure 6). Analyses of the geodetic monitoring data (there are no levelling data available before July 1999) at the benchmarks evidenced relevant vertical displacements.

The reservoir is not filled, the dam during the monitoring time suffered only clay consolidation vertical displacements, caused only by the weight of the embankment.

**Figure 7:**
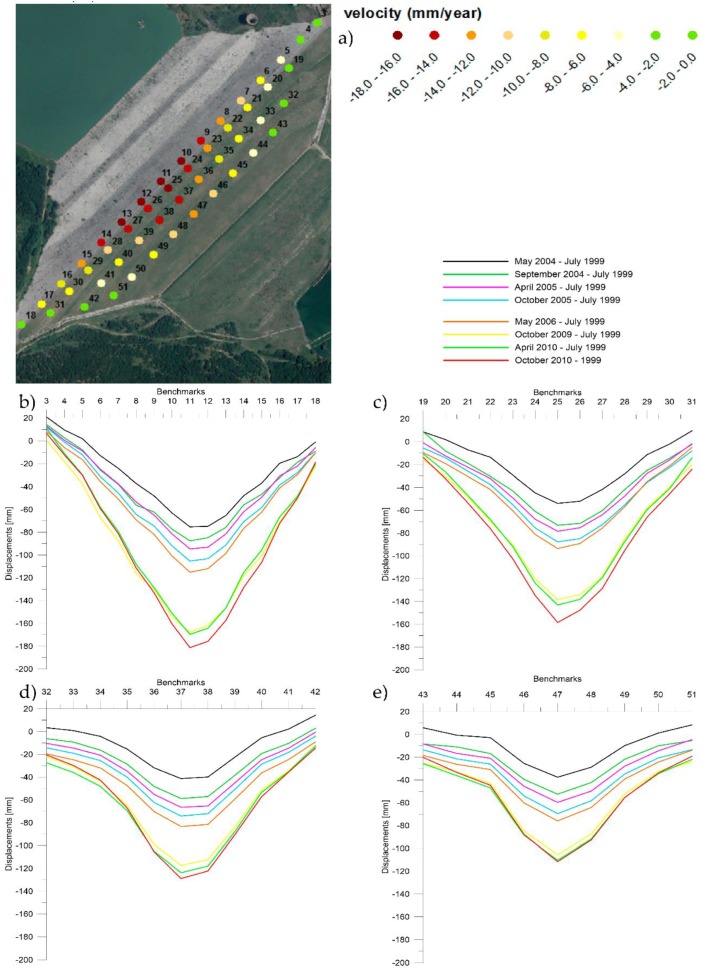
Results from the geodetic surveying: on the top, (**a**) the location of the benchmarks classified by their average velocity; bottom, cumulated displacements along the four benchmarks alignments divided for subsequent period; (**b**) from benchmark 3 to 18; (**c**) from benchmark 19 to 31; (**d**) from benchmark 32 to 42; (**e**) from benchmark 43 to 51.

Figure 7 shows that the maximum cumulated displacement is measured (from July 1999 to October 2010) along the first alignment (crest of the dam) at the benchmark 11 reaches a value of 180 mm. The graph shows similar deformation trends observed for all the alignments.

By convention, as in the geotechnical practice, deformations that lead to either a shortening of the sample or to a lowering and so subsidence of the ground level, are considered positive.

Interstitial pressure measurements are extremely useful in understanding the mechanical behaviour of the dam and for evaluating the safety conditions. The considered physical dimension is affected by the influence of various mechanical and hydraulic factors, depending on the degree of hydraulic rebalance present in the area around the measuring point. If carried out in representative points, the measurements can potentially characterize the process of increases in load, consolidation, transitory and stationary filtration. In substance, the main processes that occur during construction, the first filling and exercise and which control the evolution of the mechanical behaviour of the dam can be identified through the interstitial pressure measurements.

**Figure 8:**
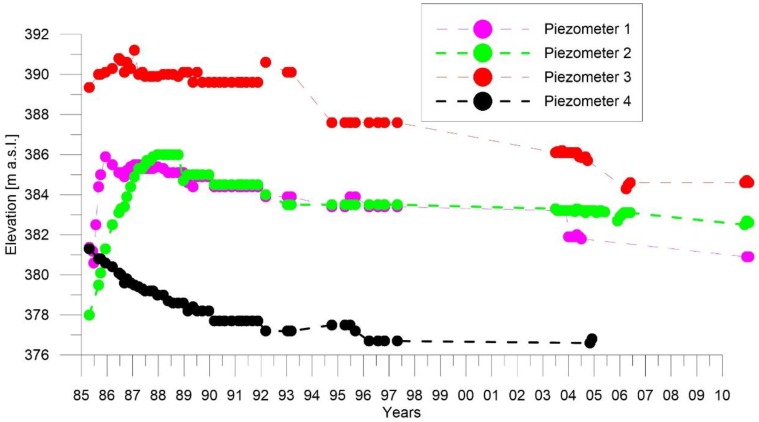
The piezometer measurements.

Figure 8 shows the evolution of the piezometric levels. The dam construction started in 1981 and was completed in 1993. The piezometers investigated, placed in the foundation of the downstream dam (P1-P2-P3-P4) showed in Figure 6 (blue squares), exhibited a pore pressure growth related with increased total stresses caused by the build-up of dam weight with a maximum pressure coinciding with the completion of the dam. After dam construction, the piezometers showed a continuous decrease due to the gradual reductions in excess pore pressure. This long-term deformation might be expected due to soil consolidation processes.

The extensometer measurements carried out both during the construction phase and during the serviceability phase show an increase in the subsidence of the foundation plan generated by the consolidation process induced on the base clays layer from the construction of the dam.

**Figure 9:**
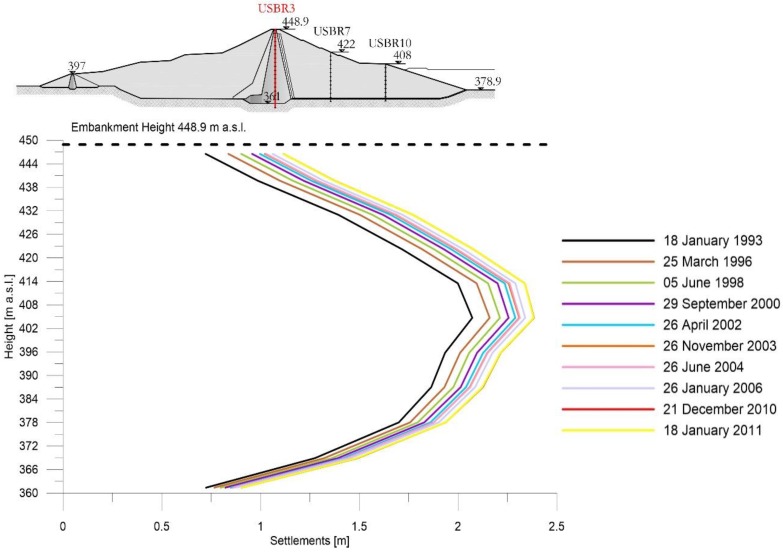
Extensometer USBR 3: settlement profiles during working phase.

**Figure 10:**
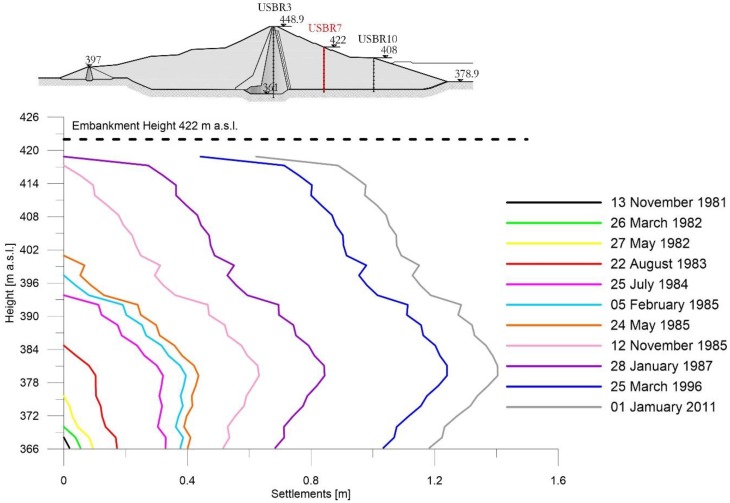
Extensometer USBR 7: settlement profiles observed during construction and working phases.

**Figure 11:**
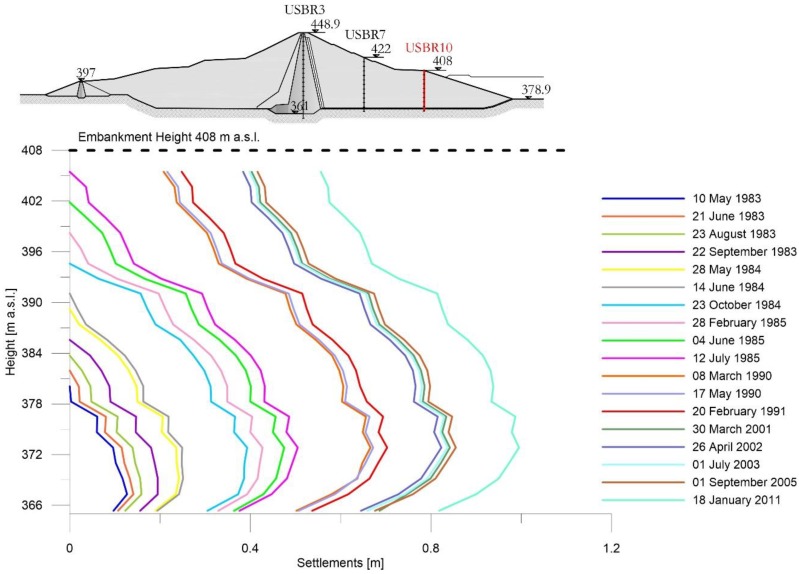
Extensometer USBR 10: settlement profiles observed during construction and working phases.

Figure 9, Figure 10 and Figure 11 show the subsidence measured respectively along the extensometric vertical A3, A7 and A10 [11].

In the Extensometer 3 (Figure 9) the available data start after the dam completion date and so they show only the consolidation process and not the building phase effect.

In the Extensometer 7 (Figure 10), the trend of the curves indicates that, after the conclusion of the embankment’s construction up to a height of 422 m a.s.l., on 28 January 1987, a rigid shift towards the positive abscissae of the assestimetric diagram is observed [12]. This being a symptom of consolidation settlement in the foundations.

In the Extensometer 10 (Figure 11), the trend of the curves indicates that after the conclusion of the embankment construction up to a height of 402 m a.s.l., on 12 July 1985, a rigid shift towards the positive abscissae of the assestimetric diagram is observed. Therefore, symptom of consolidation settlements in the foundation.

## 4. SBAS-DInSAR Technique and Dataset

The advanced DInSAR approach referred to as Small BAseline Subset [5,6] relies on a proper selection of a large number of SAR data pairs. These pairs are used to generate small baseline differential interferograms to mitigate the noise (i.e., the decorrelation phenomena) affecting the interferograms and to maximize the number of reliable points of measurement. The deformation time series are easily computed by searching for a least squares solution with a minimum norm energy constraint (the SVD technique is applied in presence of different data subsets separated by large baselines) [5]. For each coherent measure point, displacement time series and mean deformation velocity are retrieved with an accuracy of about 5–10 mm for the single deformation measurement and 1–2 mm/year for what concerns the mean deformation velocity information [7,8,9].

Moreover, the SBAS algorithm allows the generation of ground deformation time series at two different spatial scales: at the regional (low resolution) scale, it exploits average (multi-look) interferograms to detect and analyse deformation phenomena relevant to very large areas, with a ground spatial resolution ranging from about 30 to 100 m [13]. At the local (full resolution) scale, the SBAS approach exploits single-look interferograms, generated at the full sensor spatial resolution (down to a few meters), to study local deformation that may affect buildings and man-made structures [7,13,14,15]. Finally, the multi-scale SBAS algorithm also allows dealing with multi-sensor SAR data acquired by different radar systems but with the same illumination geometry, as for the case of the C-band ERS-1/2 and ENVISAT sensors [16]. To do this, the multi-sensor SBAS technique exploits only ERS-ERS and ENVISAT-ENVISAT interferometric pairs and no cross interferogram between images acquired by different SAR sensors (ERS-ENVISAT) is generated. Indeed, since the two radar sensors operate both at C-band but with slightly different signal central wavelengths, the cross-sensor ERS-ENVISAT interferograms are typically affected by severe decorrelation noise effects [17].

Thanks to the above-mentioned characteristics, the SBAS approach allows the generation of LOS displacement time series spanning very long periods (decades), thus guaranteeing the continuity in the monitoring of the Earth’s surface deformation phenomena, as well as providing unprecedented information for studying long-term ground movements at different spatial scales [18,19,20,21].

### DInSAR Dataset

**Figure 12:**
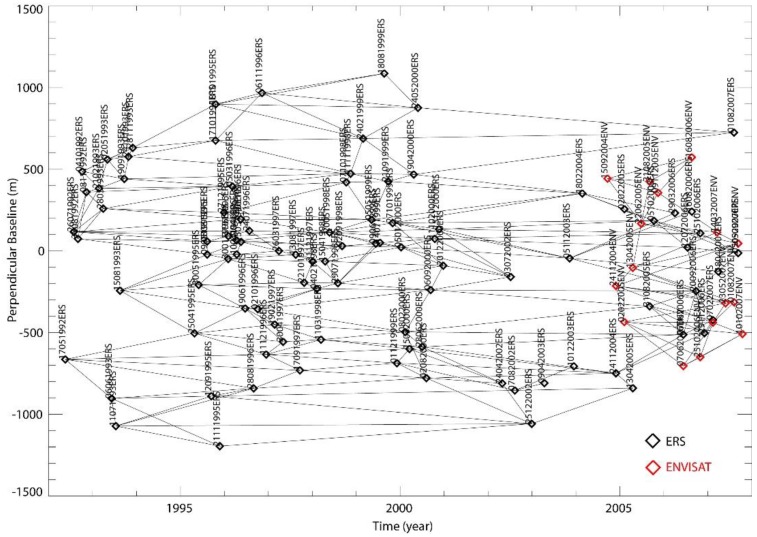
Sketch of the SAR data distribution in the temporal/perpendicular baseline plane. The black and red diamonds represent the ERS and ENVISAT acquisitions, respectively; each arc of the graph corresponds to a generated interferogram.

In this work, we applied the multi-sensor SBAS technique to a DInSAR dataset of 77 ERS-1/2 and ENVISAT scenes, acquired from descending orbits between 1992 and 2007 over the Murge area (Southern Italy). The dataset is characterized by a spatial resolution of about 7 m in slant range and 4 m in azimuth and a revisit time of 35 days. It is worth noting that, hereafter in the paper, to represent the SAR measure points we consider squared resolution cell with 10m of spatial sampling in both directions. For the SBAS-DInSAR processing, 200 ERS/ERS and ENVISAT/ENVISAT interferograms have been selected and generated (Figure 12), characterized by maximum spatial and temporal baselines of about 400 m and 1500 days, respectively. In addition, precise satellite orbital information obtained by the Delft Institute for Earth-oriented Space Re-search (DEOS) and topographic information retrieved from the Digital Elevation Model (DEM) with a 3-arc-second spacing (approximately 90 m × 90 m) produced by the Shuttle Radar Topography Mission (SRTM) in February of 2000 have been used for the correct co-registration of the DInSAR images [22] and for the interferograms generation.

## 5. DInSAR and Traditional Measurements

DInSAR data analyses, covering a period of 15 years (1992–2007) was carried out on the Genzano dam. The comparison between the results provided by the DInSAR technique and the ones from traditional methodologies usually applied for the dam monitoring is discussed in the following section. The output from the SBAS-DInSAR analysis was analysed using a GIS spatial analysis tool. Displacement velocity maps and time series were adopted to provide an overall description and to quantify the settlement between 1992 and 2007. The DInSAR measurements are only available on the crest of dam and on the upstream face, covered with rip-rap material. Due to the presence of vegetation and to the consequent lack of coherent scatterers, it was not possible to examine the downstream side of the dam.

**Figure 13:**
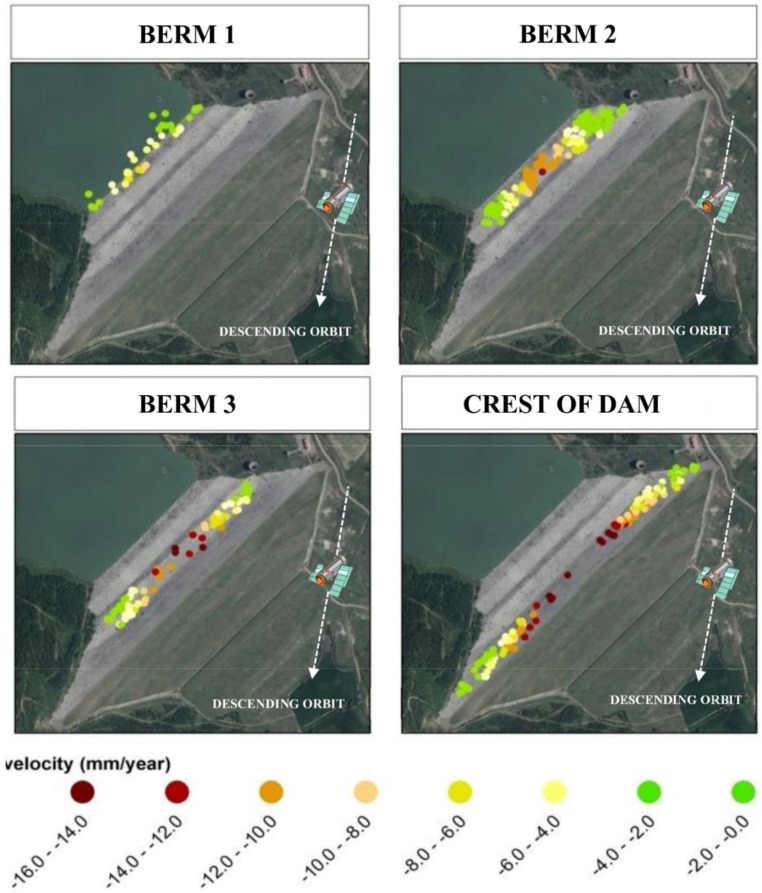
Results from the DInSAR surveying: location of the observed points classified by their average vertical displacement velocity, the white dashed line indicates the satellite descending orbit (Nord-South).

Figure 13 clearly shows that the investigated portion of the dam underwent a subsidence process, with a maximum average velocity of 15.5 mm/year as reports in the Table 3.

The displacements (almost fully ascribed to the vertical component) reached a maximum value of approximately 240 mm in the 1992–2007 period along the crest of the dam, while was quite negligible at the toe of the structure. Figure 10 shows the average vertical displacement velocity for each measurement point obtained from descending acquisition orbit. Negative velocities represent displacements that depart from the satellite (subsidence) while green colour indicates stable areas.

**Figure 14:**
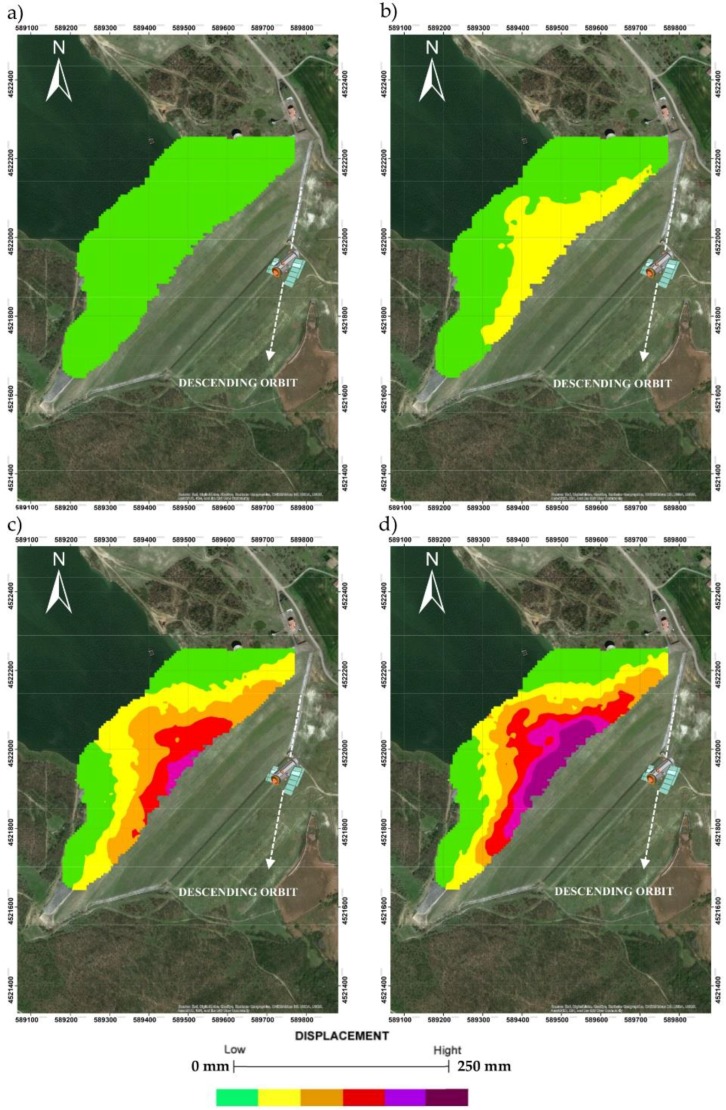
Spatio-temporal evolution of the deformation from the DInSAR observations, interpolation of punctual displacements data along the time: (**a**) 1992; (**b**) 1998; (**c**) 2002 and (**d**) 2007, the white dashed line indicates the satellite descending orbit (Nord–South).

From the punctual DInSAR data represented in Figure 13, through a Kriging interpolation process some displacements maps, reported in Figure 14, in different time have been created. In the following figures are reported the displacements observed in 1992, 1998, 2002 and 2007. The satellite observations are available since 1992 to 2007.

**Figure 15:**
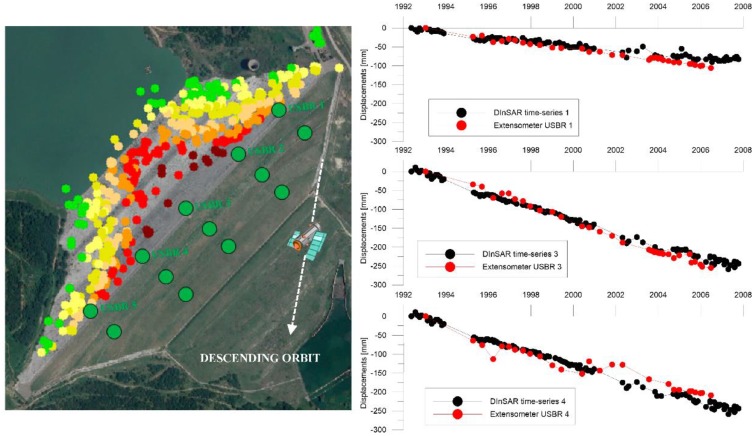
Comparison between the time-series obtained by: DInSAR (dashed black line) and in-situ extensometer measurements (red circles) on the crest of the dam, the white dashed line indicates the satellite descending orbit (Nord–South).

**Figure 16:**
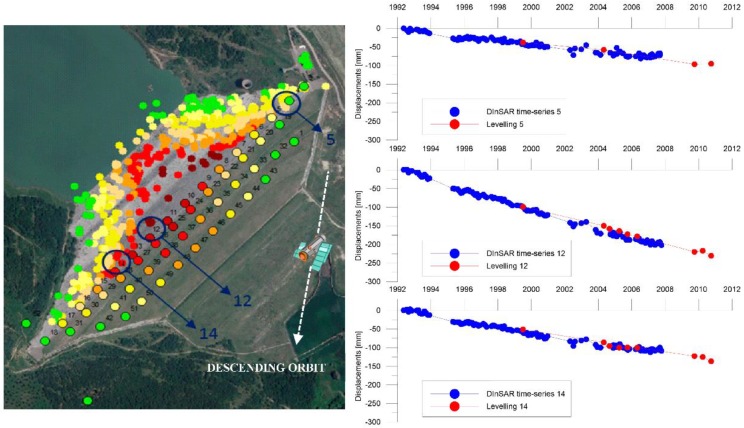
Comparison between the time-series obtained by: DInSAR (blue line) and levelling measurements (dashed red line) on the crest of the dam, the white dashed line indicates the satellite descending orbit (Nord–South).

The comparison of extensometers and levelling measurements (Figure 15 and Figure 16, Table 4 and Table 5) are in very good agreement with the DInSAR results.

As showed in the graphs in Figure 15, displacements measured by USBR1, USBR3 and USBR4 extensometers in the period 1992–2006 were compared with the time series of the corresponding DInSAR measurement points.

Similarly, levelling data collected in the period 2004–2006 were compared with DInSAR data (Figure 16). Three levelling benchmarks were selected on the crest of the dam and compared to a displacement time series obtained for the average of the most proximal DInSAR observations.

The extensometer and the spirit/optical levelling measurements show a good agreement both for each single measurement and for the long-term trends. As showed in Table 4 and Table 5, the angular coefficient of the regression trend line (i.e., the mean velocity of displacement) is very close to the values obtained from extensometers and levelling data respectively.

## 6. Geotechnical Characterization

During the design phase, several geotechnical campaigns were carried out to estimate the soil parameters such as resistance and compressibility [23].

For each survey, a geotechnical report has been drawn up and the mechanical parameters of the soil were estimated by the examination of these reports.

The lithotypes affected by the dam construction are the followings:

- grey-azure clays; and
- alluvial deposits.

The excavation to reach the construction depth of foundations is about 20 m to reach the grey-azure clays strata with best mechanical features. So, to characterize the mechanical features of the soil, it is sufficient to define the mechanical parameters of the grey-azure clays.

**Figure 17:**
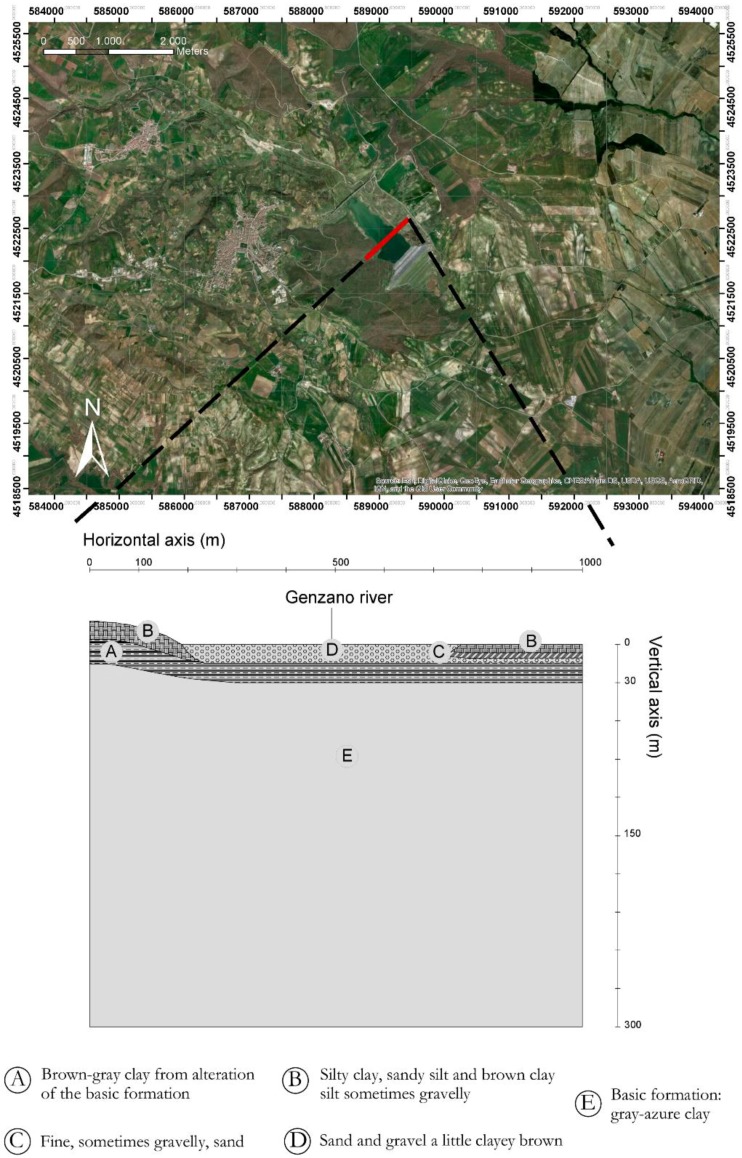
Geognostic section on the axis of the dam.

In Figure 17 a geognostic section is reported, the soil type is influenced by the presence of Genzano River, we note the presence of soils with variable granulometry, however fine, depending on the different phases of transposing and deposition of the river according to the mean sea level in the different eras.

Based on granulometric analysis, it is observed that the grey-azure clays belongs to the field of the more or less fine silt, slightly sandy, with a fair degree of compactness.

The specific weight is on average equal to 20 kN/m^3^, usual values for this kind of deposits.

The natural water content, on average equal to 18%, is close to the plasticity limit.

With regard to the characteristics of plasticity, the range of variation of the Atterberg limits is as follows:

- W_L_ = 45–70%;
- I_P_ = 28–45%.

Where W_L_ is the liquid limit calculated using the Casagrande spoon, it represents the water content for witch 25 strokes are required to close a groove made with a standardized tool on the soil placed in the spoon; and I_P_ is the plastic index obtained from the difference between liquid limit W_L_ and plastic limit W_P_. The plastic limit W_P_ is defined as the moisture content where the thread breaks apart at diameter of 3.2 mm [24].

Those values characterize a high plasticity silty clay. For carbonate content, the clays under examination can be defined as over consolidated marley clays. Regarding mechanical behaviour of the clays under examination, it has been investigated by means of edometric compression tests and triaxial compression tests of the undrained consolidated type (TRX-CU).

The edometric tests highlighted that the clays are over consolidated, both by effect of stress history and due to the cementation bonds due to the high content of carbonates present in the material.

**Figure 18:**
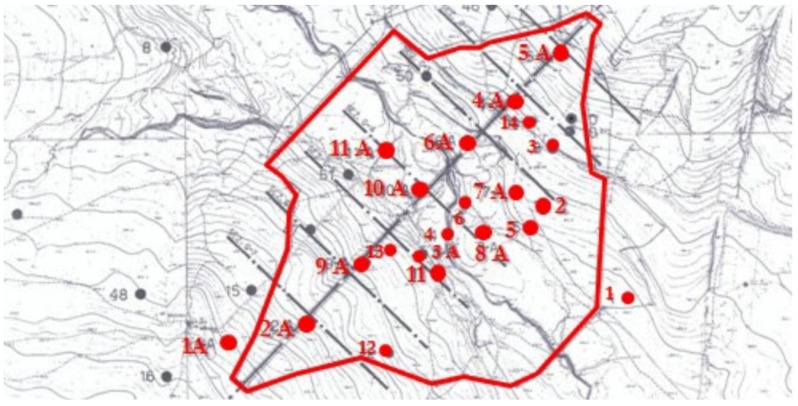
Location of collected survey samples.

In Figure 18 the location is shown of geognostic survey campaign drawn from the executive project of December 1973.

The undrained consolidated triaxial compression tests results allow the evaluation of the resistance parameters in terms of total stress:

- c’ = 150 kPa;
- φ’ = 25°.

Where c’ and φ’ are the Mohr-Coulomb yield (failure) criterion parameters, this model is often used to model soil or granular materials. This approach consists in carrying out different experimental tests with different ratios between the main stresses components, each of these tests is carried out until the limit conditions, yield or rupture, is reached drawing in the Mohr plane (σ, τ) the circle representing the relative limit stress state. The envelope of the circles thus obtained defines the plasticity limit curve; in particular, the method, approximates the most significant section of the limit curve by means of a straight line, with the following function [25,26].

$$\tau =c^{\prime }+\sigma_{n}^{\prime }\tan\varphi^{\prime }$$

The other average geotechnical features of samples, assuming the conditions as one-dimensional, analysed in laboratory drawn from the executive project of December 1973 and drawn from the tests performed during the dam construction are reported in Table 6, where are reported:

- the depth where the samples are collected;
- the specific weight γ of the collected soil;
- the water content W_n_ of each sample;
- the normal consolidation line gradient C_c_ in the plane (e, log σ’);
- the unload-reload line gradient C_S_ in the plane (e, log σ’);
- the soil permeability coefficient k; and
- the vertical consolidation coefficient c_v_.

## 7. Geotechnical Numerical Model

The present study is regarding the consolidation analysis of Genzano earth dam. Methodology in this research is based on numerical methods. Numerical analyses are carried out using the finite difference program based on a continuum finite difference discretization using the Langrangian approach [27]. By collecting the data’s such as geometric, geotechnical features of earth dam examined to investigate the consolidation process and the results are placed analysed.

In this regard, to model the dam body and its foundation the following assumptions have been taken into account based on literature suggestions [28,29]:

1. dam body has been modelled continuous, homogenous and isotropic;
2. compared to the standard section of the project, the models for the inspection and drainage tunnels downstream of the dam body and the other minor elements were excluded from the modelling;
3. the base strata of clay have an elastic modulus with increasing stiffness for deeper layers;
4. during construction, it has been assumed that the clay core has unconsolidated undrained behaviour;
5. clay core assumed to be unsaturated and going toward fully saturation on the foundation level at the end of construction;
6. the soil foundation has been divided into 5 m layers for a total of 56 layers and 280 m of thickness foundation.

To perform a two-dimensional analysis, the highest section of dam has been modelled. This section is modelled with 4400 elements.

**Figure 19:**
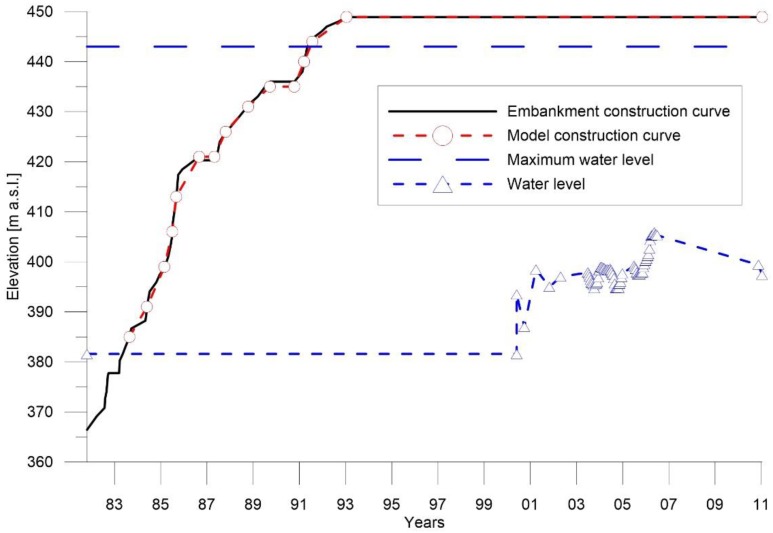
Curve construction of the dam.

The construction phases implemented in the FEM model follow the real load-history obtained from the study of original and revised works plans. In Figure 19, the black continuous line represents the planned construction curve at different works stages and the red with circle line represent the construction curve adopted in the implemented model.

**Figure 20:**
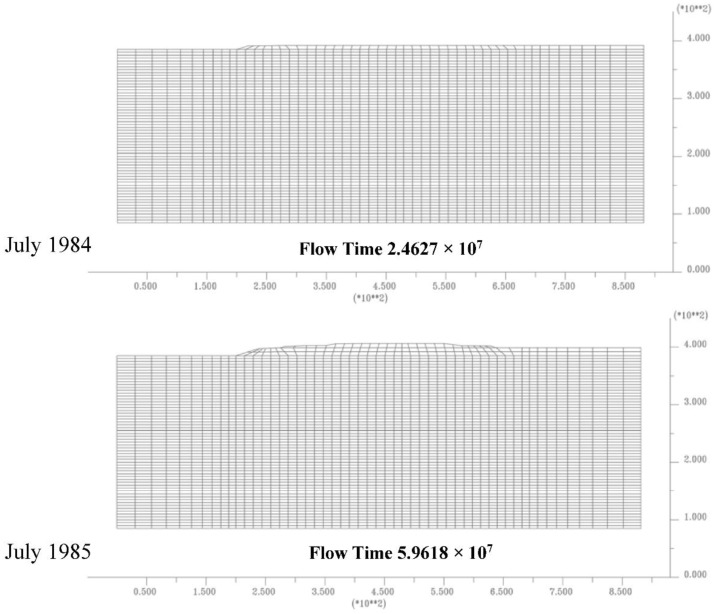
Dam’s construction steps implemented, cofferdam and first layer dam.

**Figure 21:**
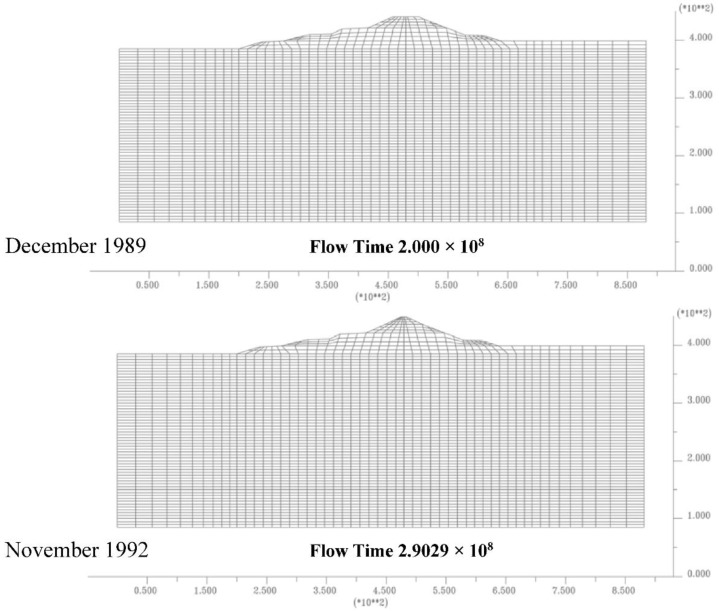
Dam’s construction steps implemented, dam realization.

Figure 20 and Figure 21 show some dam construction phases implemented inside finite element model to consider the load evolution on the soil during the time.

The DInSAR data have been used to calibrate the model parameters in order to obtain the observed displacement by means of Finite Elements Model. The following Table 7 lists the model parameters chosen after the calibration step. The bulk modulus of a substance is a measure of how the resistant to compressibility that substance is, this parameter is strictly related with the Young’s modulus E.

The permeability coefficient and compressibility coefficients (C_S_, C_C_) value are chosen on the base of consolidated triaxial shear tests performed on geotechnical samples collected in geotechnical investigations during design and construction phases.

**Figure 22:**
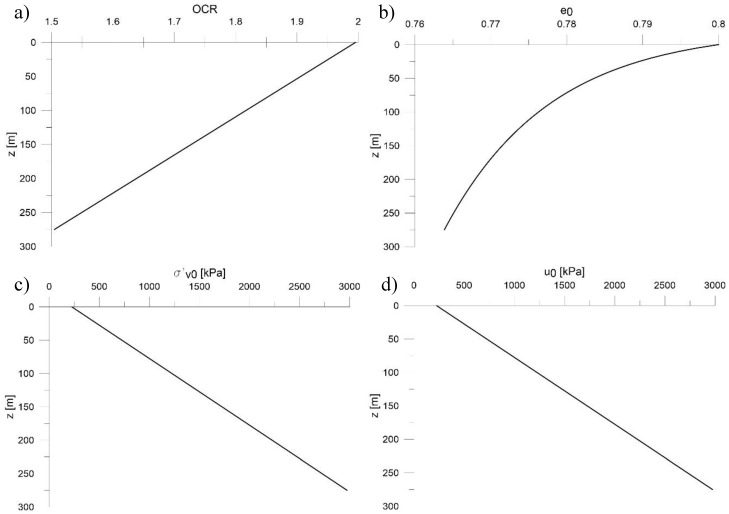
Initial conditions of modelled soil, (**a**) initial over consolidation ratio OCR; (**b**) initial void index e_0_; (**c**) initial effective vertical stress σ’_v,0_; (**d**) initial pore pressure u_0_.

The modelled soil has the following initial features, illustrated in Figure 22:

- OCR (over consolidated ratio) linear decreases according to the depth varies between 2 and 1.5, Figure 22a;
- the initial void index e_0_ decreases, as expected, according to the depth in non-linear way between 0.8 and 0.75, Figure 22b;
- the initial effective stress σ’_v,0_ varies, increasing according to the depth, between 200 and 3000 kPa, Figure 22c;
- the initial pore pressure u_0_ varies between 200 and 3000 kPa, Figure 22d.

**Figure 23:**
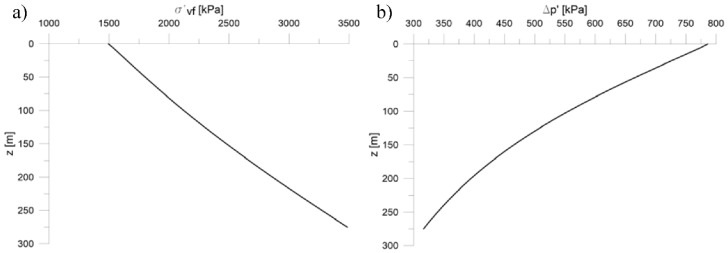
Final conditions of modelled soil, (**a**) final effective vertical stress σ’_v,f_; (**b**) increase of effective isotropic pressure Δp.’

The modelled soil has the following final features, illustrated in Figure 23:

- the final effective stress σ’_v,f_ varies, increasing depth, between 1500 and 3500 kPa, Figure 23a;
- the increase of effective isotropic pressure Δp’ varies between 300 and 800 kPa decreasing according to the depth, Figure 23b.

**Figure 24:**
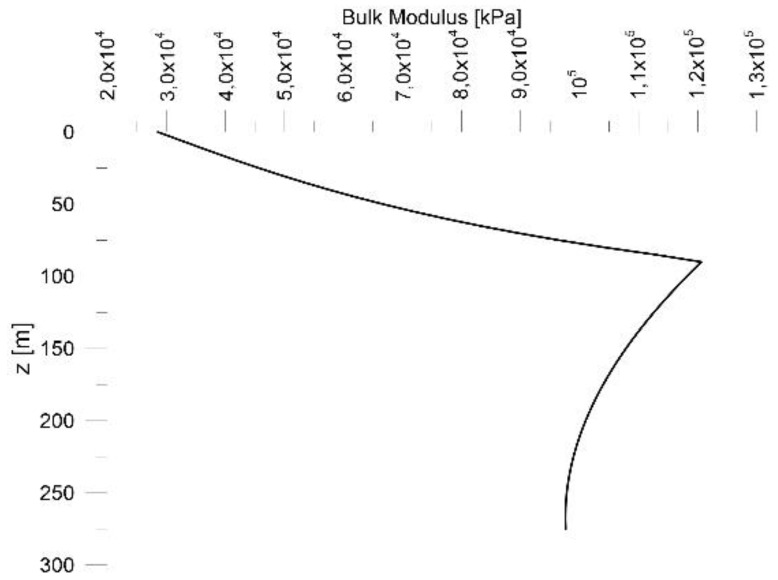
Bulk modulus K trend with depth.

The plot of Bulk modulus K variations with depth is represented in Figure 24.

## 8. Discussion of the Results

**Figure 25:**
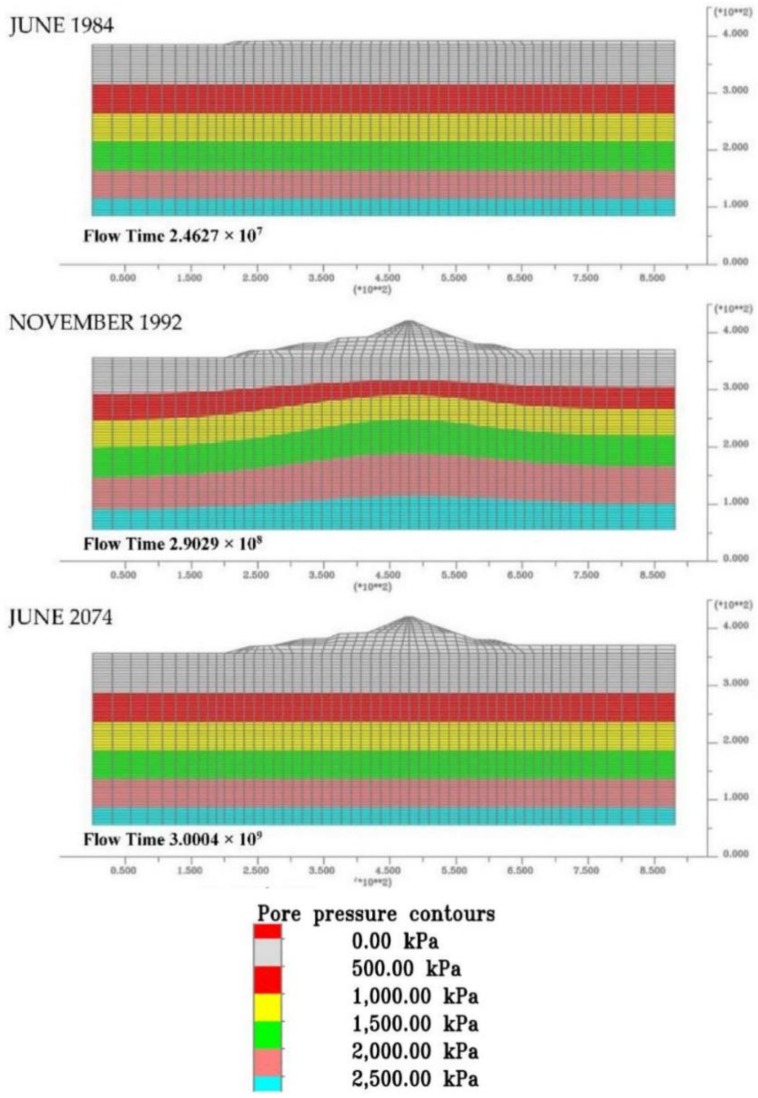
Excess water pore pressure evolution from the start of dam construction to the end of consolidation.

Some results of greater interest in terms of excess pore pressure and vertical displacements are reported for different time-steps. The contour lines of the excess pore pressure, Figure 25, exhibit a growth related to the increased total stresses caused by a build-up dam weight with a maximum pressure concurrent with the completion of the dam (1992). After dam construction, we can notice a slow exhaustion of the pore pressure, due to the consolidation process of the clay foundation, until 2074, the year in which consolidation is considered exhausted.

**Figure 26:**
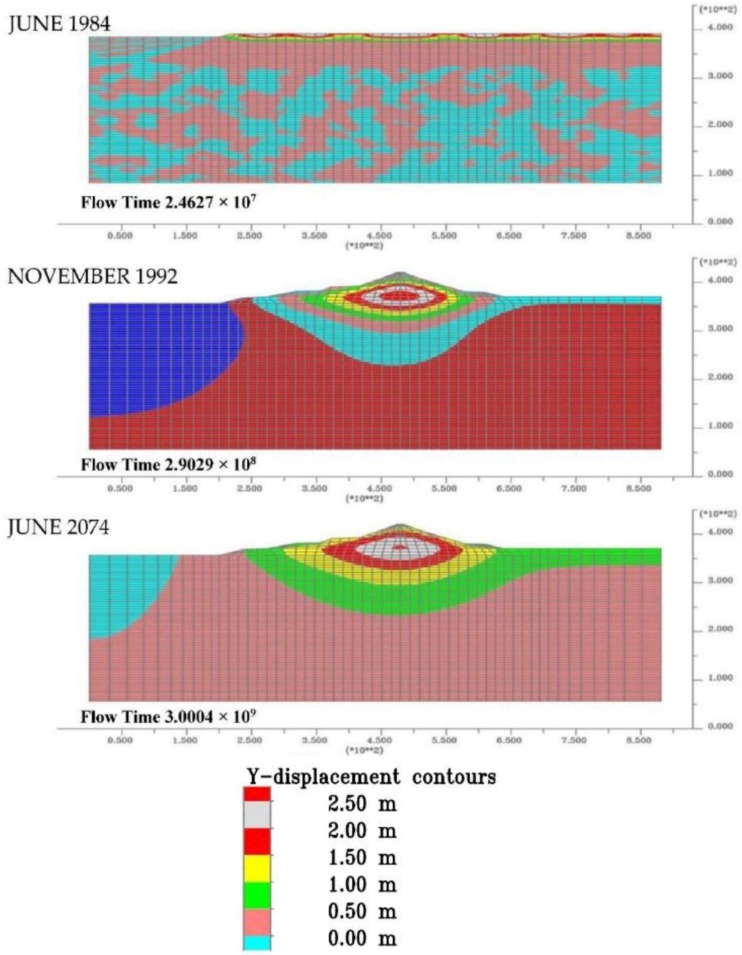
Settlement evolution from the start of dam construction to the end of consolidation.

Similar consideration mat be also done about the Figure 26 that shows the contour lines of the settlements during building phases, at the end of the construction and at the end of consolidation process.

**Figure 27:**
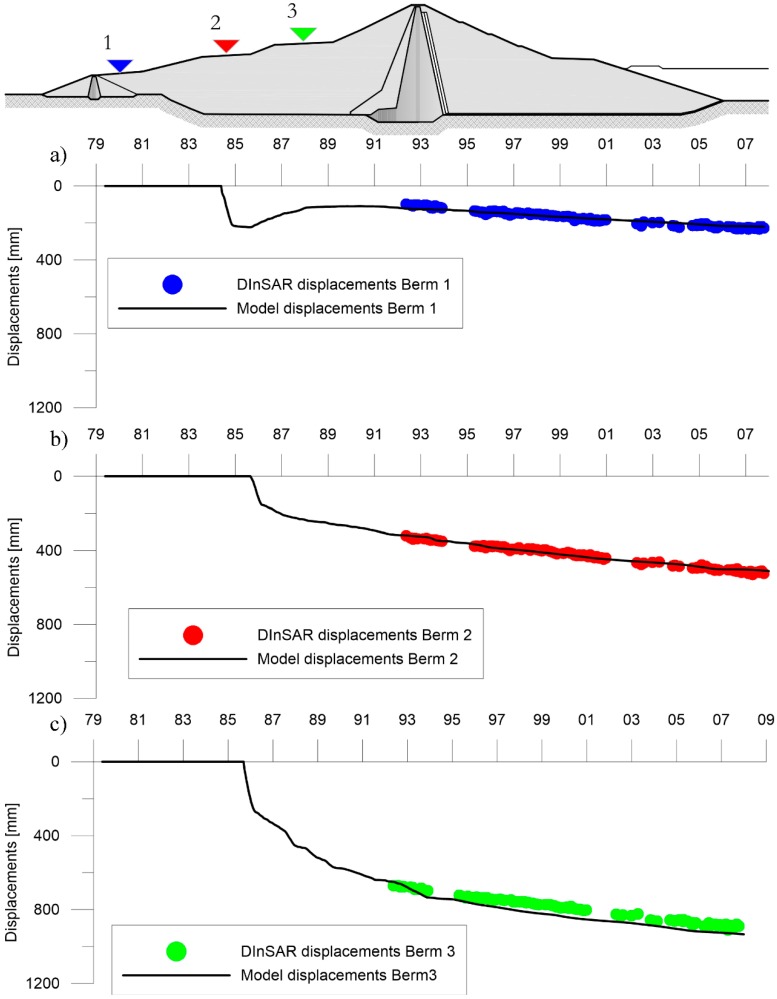
Comparison between DInSAR observed displacement and model obtained displacement for: (**a**) berm 1; (**b**) berm 2 and (**c**) berm 3.

The comparison of DInSAR observations in terms of displacements, as illustrated in Figure 27 and Table 8, are in very good agreement with the model results for each berm.

The displacement velocity reported in Table 8 was estimated for the period between 1992 and 2007 by linear regression and is to be considered in the vertical direction.

**Figure 28:**
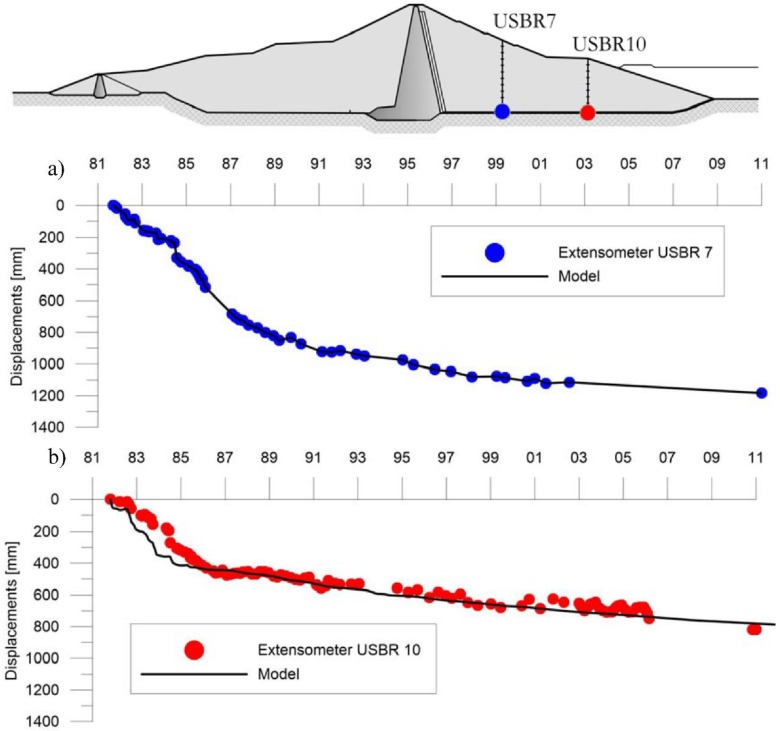
Comparison between extensometer measured displacement and model obtained displacement for: (**a**) extensometer USBR 7 and (**b**) extensometer USBR 10.

The comparison of extensometer measures in terms of displacements, as illustrated in Figure 28 and Table 8, are in very good agreement with the model results for each vertical.

The displacement velocity reported in Table 9 was estimated for the extensometer USBR 7 and USBR 10 in the period between 1981 and 2011 by linear regression.

**Figure 29:**
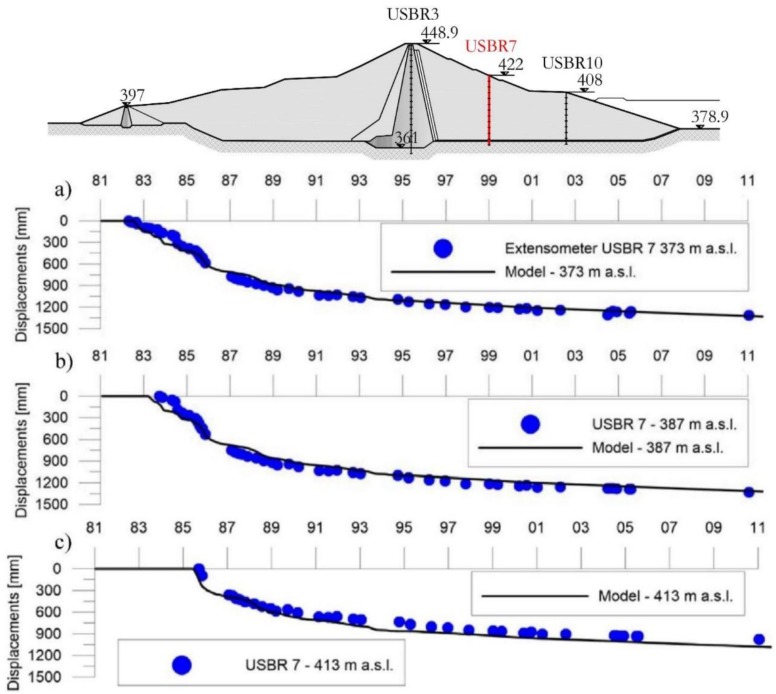
Comparison between extensometer measured displacement and model obtained displacement for USBR 7 at different heights: (**a**) 366 m a.s.l.; (**b**) 381 m a.s.l.; (**c**) 413 m a.s.l.

In the previous Figure 29 the displacements time-series are reported comparing the extensometric measures of USBR 7 at different heights, the values are in very good agreement with the model results for each height.

The displacement velocity reported in Table 10 was estimated for the extensometer USBR 7 in the period between 1995 and 2011 by linear regression.

The entire examination of the construction and consolidation process required 4000 calculation steps and approximately 45 min of processing (using a Flow Time Step of 9.508 × 10^4^ s).

The results obtained, in this case using numerical analysis, show what happens during a process of oedometric consolidation: initially the applied overload is supported only by interstitial water. Gradually the water is expelled from the pores, with vertical filtration, towards the upper and lower draining boundaries and the load is transferred to the solid skeleton that is compressed, with increase of tensions and subsidence of the clay layer. At the end of the consolidation process all overpressures have dissipated and the total overload applied is entirely supported by the solid skeleton.

The results obtained from the SBAS-DInSAR analysis have been processed in a GIS environment, where it was possible to analyse both the annual velocity maps and the deformation time series, describing the settlements observed on the dam of Genzano between 1992 and 2007. The annual average velocity map is available on the dam crest and on the upstream slope (covered with rip-rap material). On the downstream slope, there are not “coherent” points that can be observed with advanced-DInSAR technique. The DInSAR results extended for about 15 years (1992–2007), clearly show that part of the dam is subsiding (Figure 10) with a maximum average velocity of 15.5 mm/year. Results show that vertical displacements reached maximum values of 232.5 mm for the 1992–2007 period along the crest of the dam, almost exhausted, at the toe of the embankment.

The comparison between calculated and measured displacements confirmed the reliability of the implemented numerical model as well as the he reliability of the monitoring techniques.

For the elements in contact with the foundation, the displacement-time curves obtained from the Extensometers 7 and 10 and the numerical model show analogous trends (Figure 28), with an average speed of the order of 40 mm/year for the Extensometer 7 and 27 mm/year for the Extensometer 10. For the crest, a comparison was made between the historical displacement series measured from the extensometers columns 1-3-4 (the only ones from available data) and the DinSAR displacements for the period 1992–2007 (Figure 15); the results show a good correspondence between the different measurement techniques.

Finally, for the three berms placed on the upstream face (Figure 27) the comparison between the displacement-time curves obtained by numerical modelling and by DInSAR monitoring, show analogous trends and comparable average speeds (Table 8).

## 9. Conclusions

A satellite-based approach can be used to verify the provisional models of medium-long term behaviour of earth-filled dams and allows integrating the displacement data acquired by traditional techniques and spaceborne radar sensors. The data integration provides a best methodology to interpret the observed processes. Furthermore, the technique allows an instrument to calibrate the numerical model parameters used to forecast the dam’s behaviour during its life.

This work is devoted to show the capability of advanced SBAS DInSAR data as a reliable monitoring technique and to show a methodology that can be used for assessing the behaviour of a dam during life-cycle based on FEM analysis. The analysis of the time series displacements derived from SBAS-DInSAR technique reproduced the deformation behaviour of Genzano dam since 1992 allowing the performance of a back analysis in the periods in which other monitoring data were not available. Furthermore, such a long time series represented a valuable calibration dataset to set up a reliable numerical model. In order to assess the accuracy of the displacement measurements derived by SBAS-DInSAR a comparison with in-situ data was carried out showing a good agreement.

The experimented monitoring approach is mainly valuable for large dams where a sufficient number of persistent scatterers are detected by the SAR sensors. Artificial targets built on purpose can be established and positioned in the most significant parts of the structure in order to improve the performance of the method.

DInSAR techniques can be also be recommended for monitoring movements of concrete dams and surrounding slopes and, due to its very high sensitivity, it can precisely estimate the influence of various deformation sources, such as pressure of water in dammed reservoir. Using appropriate dataset, in terms of temporal and spatial resolution of data, as well as orientation of satellite line of sight towards the dam, it is possible to accurately identify dam movements, both continuously as well as periodical.

The availability of last-generation VHR images acquired by TerraSAR-X (DLR, Germany) and COSMO-SkyMed (ASI, Italy) and the large coverage C-Band data acquired by SENTINEL-1 constellation (Copernicus Programme, ESA and European Union) will hopefully allow to obtain better results in terms of ground resolution and revisiting-time by means of algorithms that produce increasingly reliable results.

## Figures and Tables

**Table 1:** Accuracy requirements of monitoring schemes.

Type of Structure	Displacement Accuracy (mm)	Recommended Monitoring Technology
Concrete dams	0.2–5	Geodetic techniques supplemented by geotechnical sensors
Embankment dams and slopes surrounding the reservoir	10–15	Geotechnical instrumentation supplemented by geodetic techniques

**Table 2:** Typical instrumentation used to monitor displacements of the dams.

Quantity Measured	Instrument
Vertical Displacements	Hydrostatic levels
	Precision topographic levelling devices
	Extensometers
	Theodolite
Angular Displacements	Clinometers
	Clinographs
Horizontal Displacements	Direct or inverted pendulums
	Theodolite
	Collimation

**Table 3:** DInSAR average vertical displacements velocity.

Position	Average Velocity (mm/year)
Berm 1	8.6
Berm 2	13.3
Berm 3	14.6
Crest of dam	15.5

**Table 4:** DInSAR vs. extensometers.

Position	Average Velocity (mm/year)	
	USBR	DInSAR
1	7.1	5.5
3	19.7	16.5
4	13.9	13.2

**Table 5:** DInSAR vs. levelling.

Position	Average Velocity (mm/year)	
	Benchmark	DInSAR
5	5.6	4.7
12	11.6	13.1
14	7.0	7.2

**Table 6:** Geotechnical features of laboratory tested samples.

Depth [m]	γ [kN/m^3^]	W_n_ [%]	C_C_	C_S_	k [m/s]	c_v_ [m^2^/s]
0–5	20.1	20.1	0.064	0.013	10^−8^	9.6 × 10^−7^
5–10	20.6	18.6	0.064	0.027	10^−8^	9.3 × 10^−7^
10–20	20.9	21.0	0.073	0.022	10^−9^	5.6 × 10^−7^
20–30	21.0	21.0	0.08	0.023	10^−11^	5.0 × 10^−7^

**Table 7:** Model parameters.

Parameters	Value
Bulk modulus K	Variable with the depth
Water Bulk Kw	2 GPa
Drained Poisson’s ratio (ν)	0.3
Permeability coefficient k	10^−11^ m/s
Unit weight (γ) (kN/m^3^)	20.0
Cc	0.076
Cs	0.032

**Table 8:** DInSAR vs. model.

Berm	Average Velocity (mm/year)	
	DInSAR	Model
1	8.7	6.9
2	14.2	13
3	14.7	19.5

**Table 9:** Extensometer vs. model.

Position	Average Velocity (mm/year)	
	USBR	Model
7	13.5	13.9
10	11.1	12.4

**Table 10:** Extensometer USBR 7 at different heights vs. model.

Height (m a.s.l.)	Average Velocity (mm/year)	
	USBR	Model
373	13.2	13.7
387	13.0	13.7
413	13.8	13.7
